# Serial crystallography for the masses?

**DOI:** 10.1107/S2052252514026803

**Published:** 2015-01-01

**Authors:** Jonathan P. Wright

**Affiliations:** a ESRF, 71 Avenue des Martyrs, Grenoble, 38000, France

**Keywords:** serial crystallography, sparse data, reconstruction of diffraction intensity, *EMC* algorithm

## Abstract

Serial crystallography should be possible with a much wider range of radiation sources as Ayyer *et al.* [*IUCrJ* (2015), **2**, 29–34] show that crystallographic intensities can be recovered from randomly oriented frames which are too sparse for indexing.

There are many important materials which do not form nice single crystals for X-ray diffraction experiments. When the crystal size is too small then powder diffraction is normally used instead. But what is the minimum crystal size needed to get three-dimensional single-crystal data? Usually this depends on instrumentation, but in this issue Ayyer *et al.* (2015 ▶) show that drastic improvements can still be made in software and algorithms.

A 100 µm crystal diffracts X-rays a million times more strongly than a 1 µm crystal, as diffracting power relates to crystal volume, not size. Signal intensity also depends on electron density and so crystals of dense heavy metals give the strongest X-ray scattering. Proteins are much more challenging; the unit cells are very large, the electron density is low, and large regions of the structure are disordered. For synchrotron radiation sources, a minimum crystal size of around 20 µm is realistic for many proteins (Sliz *et al.*, 2003 ▶) with a theoretical lower bound of about 1.2 µm for good quality lysozyme crystals (Holton & Frankel, 2010 ▶). Radiation damage is the limitation that prevents smaller protein crystals from surviving long enough to collect a full three-dimensional dataset.

Neutze *et al.* (2000 ▶) suggested that a free electron laser (FEL) would have enough X-ray flux to do away with crystals altogether. Single molecule structures should be possible provided the X-ray beam can be diffracted in the femtoseconds before the molecule is destroyed. Miao *et al.* (2001 ▶) showed the phase problem could be solved from single molecule data, and one of the first steps would be to orient many two-dimensional snapshots into a three-dimensional dataset. Similar to cryo-electron microscopy, reliably finding the particle orientation for each image needs sufficient intensity statistics. This challenge motivated Loh & Elser (2009 ▶) to invent the *EMC* algorithm. Simplistically, *EMC* assigns each two-dimensional projection the most likely orientation based on the current estimate of the three-dimensional data. The process is started by using noise for the three-dimensional data and this estimate is updated using the newly orientated projections. After several iterations the procedure can settle down on a self-consistent set of orientations. Using three-dimensional averaging gives a large boost in the signal to noise compared with pair-wise comparisons of single projections. Orientation finding turned out to be much easier for FEL data from crystals because existing software can index individual frames. The randomized orientations create a twinning problem when the crystal symmetry is lower than the lattice symmetry which Liu & Spence (2014 ▶) recently overcame using a version of the *EMC* algorithm applied to peak intensities.

In this issue, Ayyer *et al.* have applied the *EMC* algorithm to finding snap shot orientations with a known unit cell and real experimental data. Their data only needed to be precise enough to find the most likely orientation, not for complete indexing, which greatly reduces the counting statistics and number of peaks needed. Only 48 photons per frame were needed for the algorithm to converge (Fig. 1 ▶). Accumulating the snap shots directly in three-dimensional reciprocal space is also convenient for subsequent integration and processing; Yefanov *et al.* (2014 ▶) also assembled snap shot data in three-dimensional reciprocal space and showed that diffuse scattering in between the Bragg peaks can be extracted. Using a three-dimensional frame accumulation seems like it might even increase resolution limits for conventional data that are weak at high angles but can be measured with some redundancy.

Orientation finding is a ubiquitous problem in crystallography, from the use of the rotation function in molecular replacement to indexing thousands of grains in a single rotation dataset (Nervo *et al.*, 2014 ▶). In the current *EMC* algorithm an exhaustive search of all orientation space is performed and this search scales poorly with the resolution of the orientation grid. If this technique takes off then further optimizations may be expected. Ayyer *et al.* have shown the *EMC* algorithm works when the data were very weak and in a restricted orientation space. Assuming the computations will scale to full orientation space, the only problem is to collect such sparse data. This has only recently been possible with X-rays: a very fast detector which has no read noise is needed for these experiments. Having just one crystal in the beam at a time might also be difficult to confirm.

By using data from a laboratory instrument Ayyer *et al.* highlight that these revolutionary methods of serial crystallography do not need a FEL and they could make an impact on the synchrotron community in years to come. When a sample diffracts well as a powder but does not grow larger crystals, this will be a method of choice. This new way to obtain three-dimensional data could bring far more complex structures into reach for ‘powder’ diffraction.

## Figures and Tables

**Figure 1:**
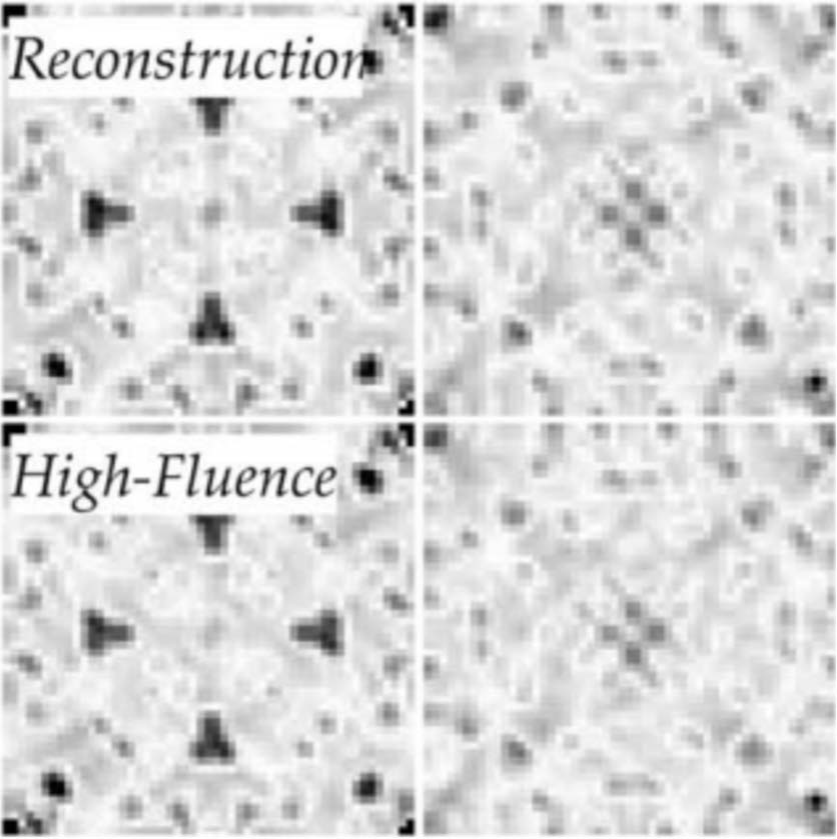
Slices of the Patterson map comparing conventional high fluence data with the *EMC* reconstruction from sparse data, which had only 48 photons per frame (from Ayyer *et al.*, 2015 ▶)
